# SSD vs. YOLO for Detection of Outdoor Urban Advertising Panels under Multiple Variabilities

**DOI:** 10.3390/s20164587

**Published:** 2020-08-15

**Authors:** Ángel Morera, Ángel Sánchez, A. Belén Moreno, Ángel D. Sappa, José F. Vélez

**Affiliations:** 1 Technical School of Computer Science, Rey Juan Carlos University, 28933 Móstoles, Madrid, Spain; a93morera@gmail.com (Á.M.); belen.moreno@urjc.es (A.B.M.); jose.velez@urjc.es (J.F.V.); 2 Escuela Superior Politécnica del Litoral, ESPOL, Guayaquil 090101, Ecuador; asappa@espol.edu.ec; 3 Computer Vision Center, Bellaterra, 08193 Barcelona, Spain

**Keywords:** object detection, urban outdoor panels, one-stage detectors, Single Shot MultiBox Detector (SSD), You Only Look Once (YOLO), detection metrics, object and scene imaging variabilities

## Abstract

This work compares Single Shot MultiBox Detector (SSD) and You Only Look Once (YOLO) deep neural networks for the outdoor advertisement panel detection problem by handling multiple and combined variabilities in the scenes. Publicity panel detection in images offers important advantages both in the real world as well as in the virtual one. For example, applications like Google Street View can be used for Internet publicity and when detecting these ads panels in images, it could be possible to replace the publicity appearing inside the panels by another from a funding company. In our experiments, both SSD and YOLO detectors have produced acceptable results under variable sizes of panels, illumination conditions, viewing perspectives, partial occlusion of panels, complex background and multiple panels in scenes. Due to the difficulty of finding annotated images for the considered problem, we created our own dataset for conducting the experiments. The major strength of the SSD model was the almost elimination of False Positive (FP) cases, situation that is preferable when the publicity contained inside the panel is analyzed after detecting them. On the other side, YOLO produced better panel localization results detecting a higher number of True Positive (TP) panels with a higher accuracy. Finally, a comparison of the two analyzed object detection models with different types of semantic segmentation networks and using the same evaluation metrics is also included.

## 1. Introduction

Although the concept of smart city (SC) was coined more than twenty years ago [1], nowadays it has a wide range of semantic interpretations and covers different meanings, which include many viewpoints of professionals and institutions involved [2]. Commonly, a SC is considered as an urban space where Information and Communication Technologies (ICT) are intensively applied to improve the quality and performance of urban services such as transportation, energy, water, infrastructures and other services (e.g., public safety) in order to reduce resource energy consumption, wastage and overall costs. The application of the best strategies, resources and available technologies to the SC environments will continuously improve the quality of life of their citizens and also the operational efficiency of these complex urban systems.

Physical and, specially, digital advertisements are becoming more common than ever in smart cities. Out-of-home (also called outdoor) advertising continues to be very effective nowadays. The deployment and maintenance of such publicity infrastructures (including their support platforms) need funds from city governments, which are mainly paid by commercial brands in order to make more visible the products and services offered. Ads have a clear impact on SCs and people notice that outdoor advertising (such as posters, billboards and digital screens) have a positive influence on them [3]. Many citizens admitted that they still have a dependence on such advertising types to know about brands and to make their buying preferences. Moreover, in their opinion, these ads contribute in making the cities appear renewed and more colorful.

The outdoor advertising industry has experienced an important growth in recent years [4]. In streets of urban environments, ads panels and billboards are everywhere, and they are also the only media that drivers and pedestrians cannot escape (i.e., differently from other forms of publicity, outdoor advertising cannot be “blocked by people”). In consequence, this is one of the most cost-effective forms of advertising available. Moreover, since current smartphones are equipped with a variety of embedded sensors like cameras, GPS or 3G/4G/5G, it is possible to get closer to the final user via a variety of Augmented Reality (AR) applications [5]. This way, the citizens using their smartphones can better develop and, perhaps, enjoy the contents associated with urban advertisements. Moreover, with the emergence of digital billboards/panels, the outdoor advertising industry is even more valuable since going digital gives advertisers the flexibility to schedule short and long-term publicity campaigns.

Advertising panels are a type of urban furniture that commonly presents a normalized shape and a more reduced size than billboards. Publicity panel detection in images offers important advantages both in the real world as well as in the virtual world. In the first case, after detection of panels, it is possible to recognize the product included in the publicity and get more information about it through AR applications. Moreover, it is possible to analyze whether or not the information of a product advertised is currently updated. In addition, a brand can use this technology to analyze the campaigns of potential competitors. Regarding the publicity on the Internet, in urban scenes, and in applications like Google Street View, it would be possible, when detecting panels on these images, to replace the publicity that appears inside a panel by another one proposed by a paying company.

**Figure 1:**
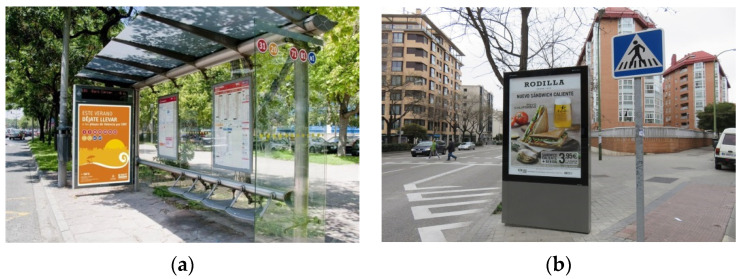
Two examples of Optical Point of Promotion and Information (OPPI) ads panels in urban outdoor images: (**a**) panel as a component of bus stop shelter; (**b**) independent panel.

In this work, we have considered the accurate and efficient detection of one specific type of outdoor advertising panel called Optical Point of Promotion and Information (OPPI). These normalized panels are commonly used in countries like France or Spain as advertising supports installed on urban furniture elements (e.g., bus stops), or located separately in busy and central places of cities. Commonly, these panels are used to hire advertising campaigns. Figure 1 illustrates these types of panels and some of the involved difficulties with outdoor images containing them.

Automatic outdoor detection and localization of OPPI ads panels (named as ‘panels’ for brevity) in real urban outdoor images is a difficult task due to multiple variability conditions presenting in the scenes containing them. For example, variable weather conditions (sunny vs. cloudy), illumination conditions (natural vs. artificial), panel perspective view, size ratio of panels with respect to image size, partial occlusions of panels or complex background in the scene (i.e., presence of multiple elements surrounding the panels like buildings, shadows, vehicles and/or different infrastructures), among other factors.

Some of the motivations of the present work are as follows: (1) accurately detecting the panels is a previous stage to analyze the content of the publicity included on them; (2) after detecting the panels it is important to classify and count the types of publicity offered by each brand in a geographical area for market prospective purposes; (3) by analyzing the contents of detected panels it is also possible to measure the “impact” of a publicity campaign; (4) for the case of “virtual” publicity on the Internet, it is possible to update the panel contents for apps like Street View or similar ones which allows providing targeted advertisements for the customers; and, finally, (5) there is an interest of companies to evaluate the quality of “physical” support of the panels to repair or substitute them. Next, we analyze the previous work related to this study and then summarize the proposed approach and its main contributions.

### 1.1. Related Work

Visual detection and recognition problems applied to specific elements in outdoor images have been previously investigated in the literature. For example, this is the case of vehicle localization [6], traffic sign detection [7] or car plates [8]. Another related problem which resembles the considered one is the detection of solar panel structures (and their orientations) in images of photovoltaic plants with no lighting restrictions, and using texture features combined with image processing techniques [9]. Some other related applications to be considered here are text and objects detection inside segmented billboard images [10] or the localization of billboards on streamed sport videos [11]. Another investigated problem is the insertion of virtual ads in street images based on localization of specific regions on them (e.g., buildings facades) [5]. Hussain et al. in [12] more recently have worked on how to build vision systems so they can understand ads, and these authors have presented a solution for automatically understanding the advertisement content.

The problem of text detection in natural scene images has also received attention in recent years (see the recent survey by Liu et al. [13]). Text detection and recognition in outdoor scenes is a key component of many content-based image analysis applications, for example the indexation of shops in a street. There are also actual conference competitions (like the one in ICDAR 2019 [14]) on the specific topic of scene text detection and recognition. Yin et al. [15] extract Maximally Stable Extremal Regions (MSERs) as character candidates which are grouped into text candidates by a clustering algorithm where parameters are learned automatically by a self-training distance metric algorithm. An effective method for scene text detection and segmentation based on cascaded Convolutional Neural Networks (CNN) is proposed by Tang et al. [16]. More recently, Xie and collaborators [17] have published a method based on Feature Pyramid Network (FPN) and instance segmentation to precisely locate text regions while suppressing false positives.

However, as far as we know, there are very few published works on detecting outdoor ads panels using a modern deep learning approach. Recently, Hossari et al. [18] have proposed the deep learning architecture ADNet, inspired in VGG19 model that automatically detects the presence of billboards with advertisements in video frames of outdoor scenes. ADNet uses the pre-trained weights of the VGG network, trained on the ImageNet dataset. After that, they re-trained the network with images of a composite dataset from Mapillary Vistas [19] and Microsoft COCO (MS-COCO) datasets [20], and achieved good test accuracy in detections.

These same authors in 2019 have also published a related work [21] for automatically detecting existing billboards in videos and replacing the advertisements contained in them with new ones. The interest is focused in learning candidate placement of billboards in outdoor scenes in order to place regularly shaped billboards in street view images. Three types of semantic segmentation networks were used in detection experiments: Fully Convolutional Network (FCN) [22], Pyramid Scene Parsing Network (PSP-Net) [23], and U-Net [24], respectively. Experimental results were evaluated using metrics derived from pixel accuracy and Intersection over Union (IoU) metrics.

Previous works on billboard detection [18,21] have considered the detection problem as a semantic segmentation one, where classification and localization was performed at the level of image pixels. Moreover, the authors have used specific deep learning networks for such a semantic segmentation task. Although semantic segmentation can be employed for the detection of billboards, from the application perspective, the annotation of images semantic segmentation is much more time-consuming, which makes it challenging for collecting large datasets. Another point is that basic detection metrics for analysis such as True Positives (TP), False Positives (FP) or False Negatives (FN) make more sense and should be redefined at the “object” level (i.e., the billboards and panels) and not at the pixel level.

Deep learning is machine learning with deep artificial neural networks [25]. The essence of deep learning is the application to learning problems of artificial neural networks that contain many hidden layers. In recent years, deep learning has been applied to many scientific domains and, in particular to image recognition problems where it has drastically improved the performance of other previous machine-learning techniques [26].

Convolutional Neural Networks (CNN) [27] are supervised shallow neural networks composed by sequences of convolutional layers followed by max-pooling layers, and so on (used for feature learning), which is followed by a fully-connected network (used for classification). Differently from previous networks like Multilayer Perceptrons where features were hand-crafted, CNN are also able to efficiently learn robust and high-level feature representations of images along the training process. Due to the impressive success of AlexNet in 2012 on the ImageNet challenge [27], CNNs have started to be used for many diverse image processing applications. AlexNet presented significant improvements upon previous image classification methods: ReLU activation function for reducing the effect of gradient vanishing during backpropagation, use of GPUs for accelerating the overall training process, data augmentation to increase the training dataset, and “dropout” (i.e., dropping out a percentage of neuron units, both hidden and visible) for reducing overfitting. In recent years, numerous deeper CNN models have appeared presenting specific refinements over previous architectures. Among these CNN-based models it is worth noting the following ones: Visual Geometry Group (VGG) networks [28] make the improvement over AlexNet by replacing large kernel-sized filters with multiple much smaller ones, one after another; GoogleNet [29] which introduced Inception layers, which can apply in parallel convolutions of different sizes to capture details at varied scales; and ResNet [30] which makes possible the stacking of layers without degrading the network performance, among others.

Object detection is a challenging task in Computer Vision that has received large attention in last twenty years, especially with the development of Deep Learning [31,32]. It presents many applications related with video surveillance, automated vehicle system robot vision or machine inspection, among many others [26,31]. The problem consists in recognizing and localizing some classes of objects present in a static image or in a video. Recognizing (or classifying) means determining the categories (from a given set of classes) of all object instances present in the scene together with their respective network confidence values on these detections. Localizing consists in returning the coordinates of each bounding box containing any considered object instance in the scene. The detection problem is different from (semantic) instance segmentation where the goal is identifying for each pixel of the image the object instance (for every considered type of object) to which the pixel belongs. Some difficulties in the object detection problem include aspects such as geometrical variations like scale changes (e.g., small size ratio between the object and the image containing it) and rotations of the objects (e.g., due to scene perspective the objects may not appear as frontal); partial occlusion of objects by other elements in the scene; illumination conditions (i.e., changes due to weather conditions, natural or artificial light); among others but not limited to these ones. Note that some images may contain several combined variabilities (e.g., small, rotated and partially occluded objects). In addition to detection accuracy, another important aspect to consider is how to speed up the detection task.

Neural-based object detectors [31] have produced, along their evolution, a state-of-the-art performance on main datasets for such a purpose. These detectors are commonly classified in two categories: two-stage detectors and one-stage detectors, respectively. The first type uses a Region Proposal Network to generate regions of interests in the first stage and then send these region proposals to the pipeline for object classification and bounding-box regression. These network models produce higher accuracy rates but are usually slower. Faster R-CNN (Region-based Convolutional Neural Networks) and Mask R-CNN are networks belonging to this group.

On the other hand, one-stage detectors handle the object detection as a regression problem by taking an input image and learning simultaneously the class probabilities and bounding box coordinates. These models initially produced lower accuracy rates but were much faster than two-stage object detectors. SSD (Single Shot MultiBox Detector) and YOLO (You Only Look Once) are included in this one-stage group.

### 1.2. Outline and Contributions of This Work

This work presents robust solutions which work at the “object” level, and using specific object detection networks for an automatic localization of panels in outdoor images. More specifically we experimented with two detectors: Single Shot MultiBox Detector (SSD) and You Only Look Once (YOLO), which were systematically compared for the considered problem. These detection networks produce rectangular windows as output with the approximate detection of each panel instance in the images together with an associate network confidence on this detection. The performance of these detectors is compared to discover the strengths and weaknesses of each one on the considered problem. For such purpose, we have properly redefined TP, FP and FN metrics at ‘panel’ level. Additional evaluation measures were used for comparison purposes. Moreover, due to the lack of available datasets of annotated OPPI panel images, we have created our own dataset which will be available for research purposes.

The paper describes a detailed experimental comparative study on the application of SSD and YOLOv3 for the considered problem in practical conditions. The main contributions of this work are the following ones:

- Experimental comparative study of deep one-stage detector networks applied to the outdoor OPPI panel detection problem. SSD and YOLO detectors are compared under multiple variability conditions (panel sizes, occlusions, rotations, and illumination conditions) to show the pros and cons of each model.
- Comparison with semantic segmentation networks for a similar problem and under the same evaluation metrics.
- Creation of an annotated dataset for this problem available to other researchers.

The manuscript is organized as follows. Section 2 introduces the materials and methods used in this research on detection of outdoor ads panels. Section 3 describes the experimental setup and presents the results achieved for the considered problem. Section 4 analyzes and discusses these results. Finally, in Section 5 we summarize the conclusions of the work.

## 2. Materials and Methods

In this section we describe in detail the considered one-stage detection models: SSD and YOLOv3, respectively. An overview of the stages in the proposed solution is presented. The panel image pre-processing stage is next explained. We continue with the parametrization of the two detectors considered for the specific problem, and also include some details about training these networks. Finally, the dataset used in experiments is briefly described.

### 2.1. SSD and YOLOv3 Models

**Figure 2:**
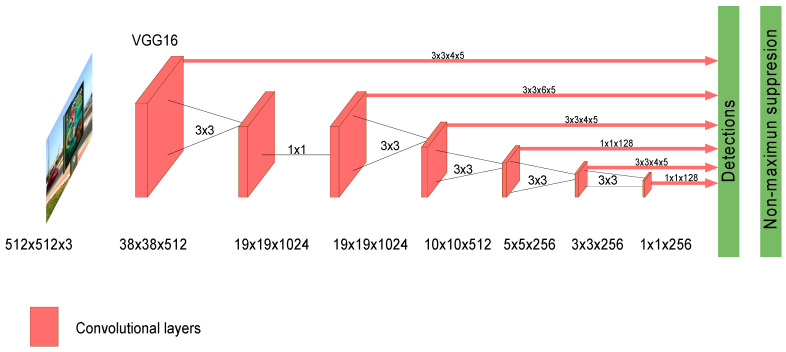
Layer architecture of Single Shot Multibox Detector (SSD) network.

The Single Shot MultiBox Detector (SSD) network was proposed by Liu et al. in 2015 [33]. SSD introduces multi-reference and multi-resolution detection techniques. Multi-reference techniques define a set of anchor boxes of different sizes and aspect ratios at different locations of an image, and then predict the detection box based on these references. Multi-resolution techniques allow detecting objects at several scales and at different layers of the network. A SSD network implements an algorithm for detecting multiple object classes in images by generating confidence scores related to the presence of any object category in each default box. Moreover, it produces adjustments in boxes to better match the object shapes. This network is suited for real-time applications since it does not resample features for bounding box hypotheses (like in models such as Faster R-CNN [34]). The SSD architecture is CNN-based and for detecting the target classes of objects it follows two stages: (1) extract the feature maps, and (2) apply convolutional filters to detect the objects. SSD uses VGG16 [28] to extract feature maps. Then, it detects objects using the Conv4_3 layer of VGG16. Each prediction is composed of a bounding box and 21 scores for each class (one extra class for no object); the class with highest score is selected as the one for the bounded object. Conv4_3 makes a total of 38 × 38 × 4 predictions: four predictions per cell independently from depth of feature maps. Many predictions will contain no object as it is expected and uses the class ‘0’ to indicate that no object was detected in the image. Figure 2 illustrates the typical layer structure of a SSD network.

Regarding the objective loss function, SSD proposes to use a weighted sum of the localization loss (*loc*) and the confidence loss (*conf*). Let $x_{ij}^{p}=\left\{0,1\right\}$ be an indicator for matching the *i*-th default box to the *j*-th ground truth box of category *p*, the overall objective loss is defined as:

(1) $$L\left(x,c,l,g\right)=\frac{1}{N}\left(L_{conf}\left(x,c\right)+\alpha L_{loc}\left(x,l,g\right)\right)$$

where *N* is the number of matched default boxes. The $L_{loc}$ is a L1 loss between the predicted box (*l*) and the ground truth box (*g*) parameters. SSD regress two offsets for the center (*cx*, *cy*) of the default bounding box (*d*) and for its width (*w*) and height (*h*):

(2) $$L_{loc}\left(x,l,g\right)=\sum_{i\epsilon Pos}^{N}\sum_{m\epsilon \left\{cx,cy,w,h\right\}}x_{ij}^{k}smoth_{L1}\left(l_{i}^{m}-{\hat{g}}_{j}^{m}\right)\;{\hat{g}}_{j}^{cx}=\frac{\left(g_{j}^{cx}-d_{i}^{cx}\right)}{d_{i}^{w}}\;{\hat{g}}_{j}^{cy}=\frac{\left(g_{j}^{cy}-d_{i}^{cy}\right)}{d_{i}^{h}}\;{\hat{g}}_{j}^{w}=\log\left(\frac{g_{j}^{w}}{d_{i}^{w}}\right)\;{\hat{g}}_{j}^{h}=\log\left(\frac{g_{j}^{h}}{d_{i}^{h}}\right)$$

The confidence loss is the *softmax loss* over multiple classes confidences (*c*):

(3) $$L_{conf}\left(x,c\right)=-\sum_{i\epsilon Pos}^{N}x_{ij}^{p}\log\left({\hat{c}}_{i}^{p}\right)-\sum_{i\epsilon N\;eg}\log({\hat{c}}_{i}^{0})\;where\;{\hat{c}}_{i}^{p}=\frac{\exp(c_{i}^{p})}{\sum \exp(c_{i}^{p})}$$

and the weight term ∝ is set to 1 by cross validation.

A You Only Look Once (YOLO) detector was proposed by Redmon et al. in 2016 [35] and it is oriented to real-time processing. YOLO was inspired by GoogleNet and the idea was applying a unique neural network to the full image, where the network divides the image into regions and simultaneously predicts bounding boxes and probabilities for each region. These bounding boxes are weighted by the predicted probabilities. YOLO splits an image into a *N* × *N* grid, where each cell predicts only one object. This prediction is given as a fixed number of boundary boxes where each box has its confidence score. It detects one object per grid cell regardless of the number of boxes by applying a non-maxima suppression algorithm. YOLO generally uses ImageNet for parameter pre-training, and then uses target detection data sets for target recognition training. Several improvements on YOLO architecture have been proposed (i.e., YOLOv2 and YOLOv3 versions) which increased the detection accuracy while keeping a very high detection speed.

**Figure 3:**
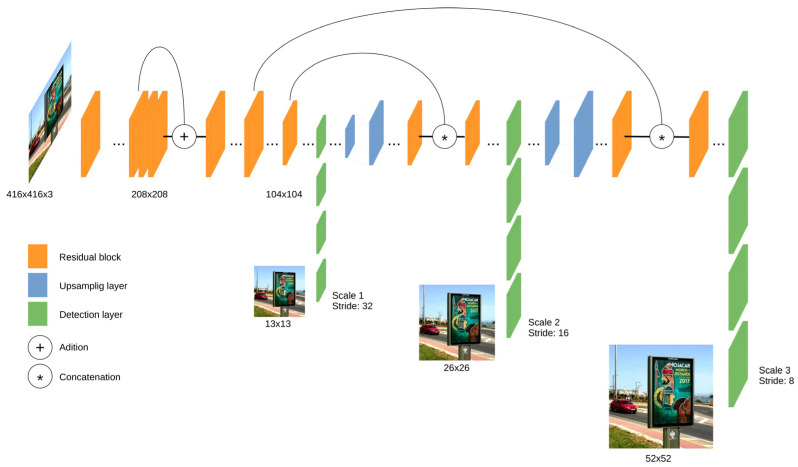
Simplified layer architecture of You Only Look Once (YOLO)v3 network.

YOLOv3 [36] uses a variant of Darknet architecture and has 53 layers trained with the ImageNet dataset. For the object detection tasks, an additional 53 layers were added, and this model was trained with the Pascal VOC dataset. YOLOv3 outperformed most of the detection algorithms for real-time applications. Using residual connections and upsampling, the architecture can perform detections at three different scales from the specific layers of the structure. This makes YOLOv3 model more efficient when detecting small objects but, on the other side, it results in slower processing than the previous versions due to the complexity of the solution. Figure 3 shows a simplified layer structure of YOLOv3.

The strategy followed by YOLO is as follows. First, it divides the given image into an *S* × *S* grid. Then, each grid cell is used to analyze whether an object falls into it or not. Hence, each grid cell predicts *B* bounding boxes and confidence scores for those boxes. These confidence scores reflect how confident the model is that the box contains an object and also how accurate this prediction is. Each bounding box consists of 5 predictions: $d^{cx},\;d^{cy},\;d^{w},d^{h}$ (i.e., bounding box center coordinates and its width and height) and confidence. For each grid also the cell conditional class probabilities $c_{i}^{p}$ is predicted. In summary, the loss function is defined as:

(4) $$Loss=\lambda_{coord}\sum_{i=0}^{S^{2}}\sum_{j=0}^{B}\Pi_{ij}^{obj}[{\left(d_{i}^{x}-{\hat{d}}_{i}^{x}\right)}^{2}+{\left(d_{i}^{y}-{\hat{d}}_{i}^{y}\right)}^{2}]\;+\lambda_{coord}\sum_{i=0}^{S^{2}}\sum_{j=0}^{B}\Pi_{ij}^{obj}[{\left(\sqrt{d_{i}^{w}}-\sqrt{{\hat{d}}_{i}^{w}}\right)}^{2}+{\left(\sqrt{d_{i}^{w}}-\sqrt{{\hat{d}}_{i}^{w}}\right)}^{2}]\;+\sum_{i=0}^{S^{2}}\sum_{j=0}^{B}\Pi_{ij}^{obj}{\left(c_{i}^{p}-{\hat{c}}_{i}^{p}\right)}^{2}\;+\lambda_{noobj}\sum_{i=0}^{S^{2}}\sum_{j=0}^{B}\Pi_{ij}^{noobj}{\left(c_{i}^{p}-{\hat{c}}_{i}^{p}\right)}^{2}\;+\sum_{i=0}^{S^{2}}\Pi_{i}^{obj}\sum_{c\;\epsilon \;classes}{\left(p\left(c_{i}^{p}\right)-\hat{p}\left(c_{i}^{p}\right)\right)}^{2}$$

where $S^{2}$ is the output feature map of all grid cells, *B* is the number of bounding box for each grid, *i* is the *i*-th grid, *j* is the *j*-th predicted box of this grid, *obj* is with object, *noobj* is no objects, *c* is the confidence of real objects, $\hat{c}$ is the confidence of predicted objects, $p\left(c_{i}^{p}\right)$ is the probability of real box category, $\hat{p}\left(c_{i}^{p}\right)$ is the probability of predicted box category, $\Pi_{i}^{obj}$ denotes if object appears in cell *i* and ($\Pi_{ij}^{obj},$ $\Pi_{ij}^{no\;obj}$) judges whether the *j*th box in the *i*th grid is responsible for that prediction, and ($\lambda_{coord}$, $\lambda_{no\;obj})$ are weighting factors.

### 2.2. Proposed Solution

**Figure 4:**
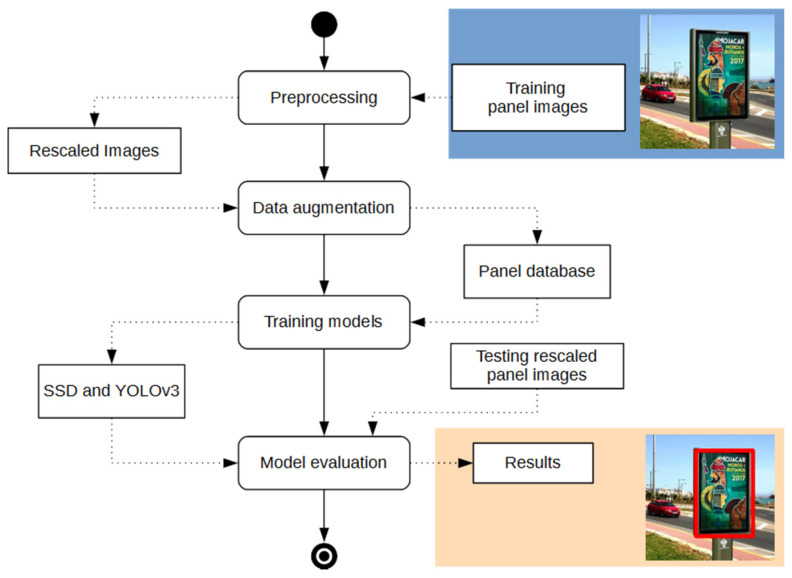
Overview of proposed method for panel detection.

Figure 4 shows a UML diagram illustrating the stages followed in the proposed solution for both SSD and YOLOv3 models. First, the original set of training images was preprocessed and augmented to increase the size of the training dataset. Preprocessing included an image rescaling to adapt it to the respective sizes of the input layers of SSD and YOLOv3 networks. The neural architectures were trained, tested and compared using the same dataset and under the same evaluation metrics.

### 2.3. Image Pre-Processing and Data Augmentation

One type of image preprocessing consisted in rescaling the original images by preserving their aspect ratio and using the sizes of respective input layer for SSD and YOLOv3 networks. For such purpose, in the SSD model, the shorter side of an image was set to 512 pixels and the larger side was set to the proportional size in pixels, so that the aspect ratio was preserved. After that, the larger dimension of the image was trimmed so that it would also be 512 pixels (i.e., spatial resolution of 512 × 512) without losing any part of the panel. Original images for the YOLOv3 network were analogously preprocessed to a resolution of 416 × 416 pixels.

After that, we applied data augmentation for training using the tools provided by the DarkNet neural network framework [36]. It allows different types of geometric and color transformations to be applied to the images. For example, image scalings, rotations and transforming the colors of the image based on saturation, exposure and hue values. In our case, since the number of original training and validation images was small and the dataset was also unbalanced with respect to variabilities present in the panels and in the images, a data augmentation stage was applied to balance this dataset and to increase the dataset size. For such a purpose, we took original patterns from “opposite” variability classes with a lesser number of elements (i.e., “oblique panels”, “occluded panels” and “night images”, respectively), and we applied to them some slight rotations (between −5° to 5°) and zooms on the images (from −10% to 10%) to increase the size and variability of our dataset. This augmentation produced a larger dataset containing 5884 training and validation images, which multiplied the number of original images by about three.

### 2.4. Parametrization of Network Detectors and Training Details

SSD training needs a collection of input images and their corresponding ground truth boxes for each class object contained in them. In our approach, a SSD MobileNet v1, pre-trained with Microsoft COCO dataset [20], was used. MobileNets [37] are a family of more efficient neural models including depth-wise separable convolutions, suitable for mobile and embedded vision applications. The network input was adapted to the size of our preprocessed images. Then, it was finely tuned and trained using our own panel dataset (some details on the dataset are given in next subsection). In our problem, only one class was required (i.e., the ‘panel’ class) and the network itself can discriminate in the images between what is a ‘panel’ and what is not. Experiments were performed using a small batch size of between 6 and 10, and different numbers of epochs up to 176,000. RMSProp algorithm was used as optimizer. Different values of learning rates varying from 0.001 to 0.004 were evaluated, with a momentum of 0.9. Approximately, a number of 5900 images with panels were used for training and validation of both SSD and YOLOv3 networks.

To train the YOLOv3 network the code of Darknet project was adapted. Darknet [36] is an open source neural network framework written in C and CUDA. This framework was pre-trained using the ImageNet dataset [27]. After that, we adapted the weights of this pre-trained model to our one-class detection problem, and trained this network using our set of labelled images. These input images for training have been re-scaled to a spatial resolution of 416 × 461 × 3 (RGB images) using the pre-processing described in the previous subsection. The training was carried out during 5000 iteration cycles and it used the optimizer SGD Burn-In of Darknet, with learning rate values varying from 0.0001 to 0.01, and the momentum was between 0.8 and 0.9. The number of max-batches and the size of the batches were set to 4000 and between 4 and 8 images, respectively.

Table 1 summarizes some important training parameters used for SSD and YOLOv3 in our experiments.

As mentioned in Section 2.1, regarding the loss function, for the case of the SSD network a combination of two criteria was employed: classification and regression loss, respectively. Classification loss measures the confidence level in the predictions of each bounding box returned by the network. This loss is computed using Categorical Cross-Entropy. Regression loss measures the distance between the bounding boxes predicted by the network with respect to the real bounding boxes of the ground truth. The L2-Norm measurement is used for this purpose.

In the case of YOLOv3 network, the loss function is computed for each of the three scales of the architecture. Each scale used 85 dimensions to calculate the loss. The first four dimensions correspond to *x*-center coordinate, *y*-center coordinate, height and width of bounding box, respectively. The fifth dimension corresponds to objectness confidence score of the bounding box. The last 80 dimensions correspond to the predicted classes (in our case, we only consider the “panel” class). Four types of loss are computed: (1) MSE (mean squared error) of x-center, y-center, height and width of bounding box; (2) BCE (Binary Cross Entropy) of objectness score of a bounding box; (3) BCE of no objectness score of a bounding box; and (4) BCE of multi-class predictions of a bounding box, respectively.

All of our algorithms were coded in Python using the OpenCV Computer Vision library and the Keras high-level API for neural networks. These codes and related information about the project can be downloaded from: https://github.com/jfvelezserrano/ads_panel_detection. All our models were trained and tested using an Intel(R) Core(TM) i7-7700HQ CPU@2.80 GHz, 8 GB RAM, GPU GeForce^®^ GTX 1050 with 2 GB. Average detection times of panel(s) per image were 200 ms for SSD and 80 ms for YOLOv3, respectively.

### 2.5. Description of the Used Dataset

We have not found any publicly available dataset of outdoor urban panel images with the characteristics we are considering for our study (i.e., the corresponding ones to OPPI panels). A related referenced dataset of billboard adverts is CASE (CAndidate Spaces for advErt implantation) [21] which was built from the Cityscapes dataset [38] and includes street view images. The CASE dataset was created by randomly selecting 10,000 images from Cityscapes dataset, and annotating them with the placements of advertisements. However, this dataset is not currently available.

Consequently, we created our own dataset of the considered type of panels in order to train the detection networks to be evaluated and then compared: SSD and YOLOv3, respectively. This dataset will be released to other researchers interested in the considered problem. We have firstly collected approximately 1800 images of these panels (both from the Internet and also by taking photos of them), which were separated into training and validation sets. Additionally, a number of 261 test images were collected separately, and they contained a number of 283 panels in total. Because of the number of training images was small and the dataset was also unbalanced with respect to variabilities present in the panels and in the images, a data augmentation stage (as described in Section 2.4) was applied to balance this dataset and to increase the sample size. This augmentation produced a dataset with 5884 training and validation images. More precisely, for the SSD model 5400 images were used for training and the 484 remaining ones for validation; for the YOLOv3 network 5295 and 589 were, respectively, used for training and validation. All of the training, validation and test images were manually labeled (i.e., by marking two opposite rectangle points per panel) using the VGG Image Annotator Tool [39] in order to produce the ground-truth regions where panels were located in the images. Next, the annotated information in each image was stored and adapted to the TensorFlow API. Note that all of the considered images were from outside and they contain at least one publicity panel (some of them contained more than one).

Table 2 shows the distribution of considered panels in the test images according to the four types of variabilities being analyzed: panel size ratio, panel orientation in image (frontal vs. oblique), panel occlusion (non-occluded vs. partially occluded) and scene illumination (day vs. night images), respectively.

**Figure 5:**
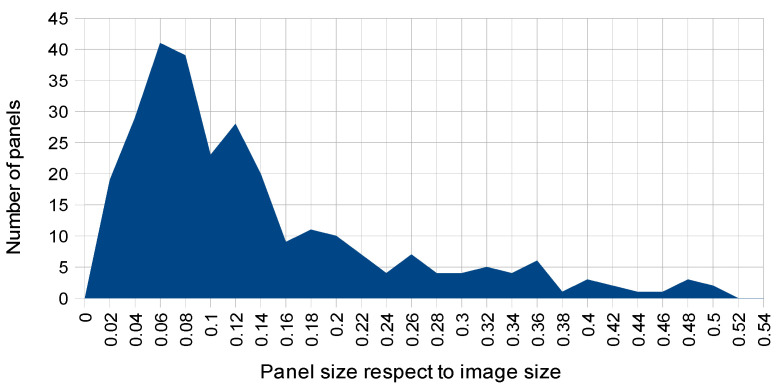
Histogram of panel coverage sizes in test images.

A histogram with the detailed distribution of panel sizes is shown in Figure 5. Note that in our test dataset there are no panels covering more than half of the image. Regarding the other considered variabilities the two types of panel positions (frontal vs. oblique) are not too unbalanced, as it is the case with respect to occlusions (most of the test panels are not occluded) and illumination (most of panel images were captured with daylight illumination).

**Figure 6:**
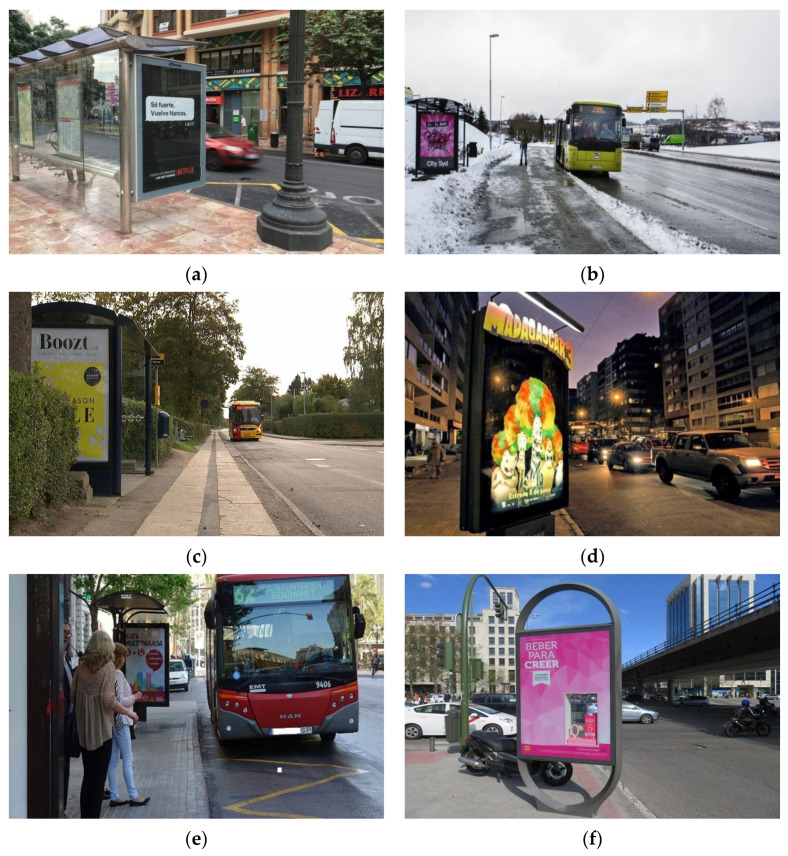
Test images of panels including some considered variabilities: (**a**) rotated panel; (**b**) reduced size ratio with respect to image size (smaller than 10%); (**c**) partial occlusion of panel; (**d**) night image; (**e**) combined reduced size and partial occlusion of panel; (**f**) combined rotated panel with “window”.

Figure 6 shows several sample test images corresponding to some types of variabilities considered in our dataset. Note that some scenes containing panels can present several types of these variabilities at the same time. For example, the images (e) and (f), respectively, illustrate two examples of possible combined variabilities in the OPPI panel scenes.

## 3. Results

In order to evaluate our approach, we use different standard performance metrics related to the quality of detections produced by the compared SSD and YOLOv3 networks. First, we explain the basic accuracy measures in the context of our problem. Next, we use the Intersection over Union (IoU) and F1-score to evaluate the accuracy in the detection of panels. After that, we show the detection results for SSD and YOLOv3 under the different types of variabilities and compare them. In order to compare our results with those presented in the work by Dev et al. [21], we introduce and apply the same additional measures used by these authors. Finally, we present a global discussion on results achieved in the work.

### 3.1. Description of Performance Metrics

To define basic accuracy measures over the detections, it is necessary to consider the following threshold parameters: network confidence loss threshold and IoU threshold, respectively. Network confidence loss is returned by the detector, and it measures how confident the network is of the objectness in the computed bounding box. Categorical cross-entropy is used to compute this loss. IoU measures how accurately an object is detected in a test image. A confidence threshold *Conf_th_* is used to determine a network gives a positive answer relative to a detected object in the image. An IoU threshold *IoU_th_* is used to determine that overlapping between network detection and the ground truth is significant. After some experimentation, we set the value of these parameters to *Conf_th_* = 0.5 and *IoU_th_* = 0.6, respectively.

Basically, the results produced by our one-stage detector networks consist of a collection of rectangular windows corresponding to each detected object in the image, and for each window it is also returned the object class corresponding to the detection and its confidence loss.

In the context of our panel detection problem on images, it is necessary to redefine the True Positive (TP), False Positive (FP), True Negative (TN) and False Negative (FN) in relation to the detections produced on the images. If Conf(*p*) is the confidence loss returned by the network on the detection of the panel *p* present in image *i*, and IoU(*p*) is the intersection over union value for the same panel, then *p* is considered as a TP, FP, TN or FP when any of the following conditions hold:

TP(*p*) = (Conf (*p*) >= Conf_th_) AND (IoU (*p*) >= IoU_th_) (5)

FP(*p*) = (Conf(*p*) >= Conf_th_) AND (IoU(*p*) < IoU_th_) (6)

TN(*p*) = (Conf(*p*) < Conf_th_) AND (IoU(*p*) < IoU_th_) (7)

FN = NP(*i*) − |TP(*i*)| (8)

In FN, the network does not give any confidence, NP(*i*) and |TP(*i*)| represent the number of panels present in the image *i* and the number of TP in the same image, respectively. Note that FN condition is computed at the level of the image. Moreover, we also accumulate the numbers of TP, FP and FN detections for each image *i*, and also for the whole dataset to present and compare our test results for SSD and YOLOv3. For simplicity, we also denote these accumulated values of TP, FP and FN in the whole dataset in this form.

**Figure 7:**
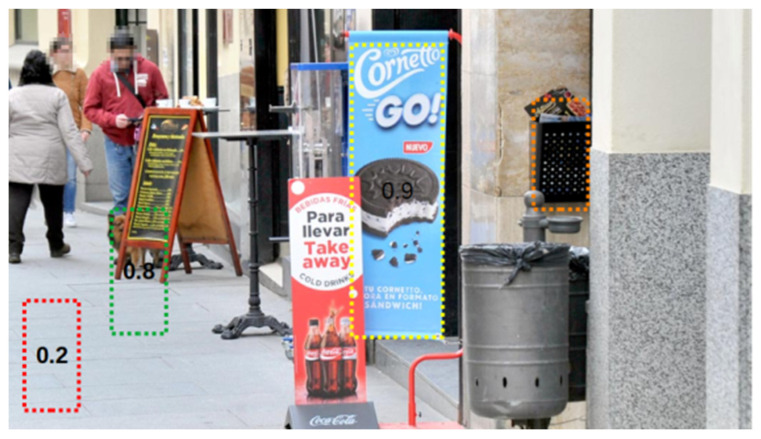
Illustrative image to show accumulated True Positive (TP), False Positive (FP), True Negative (TN) and False Negative (FN). Dotted rectangles in different colors represent the detections produced together with the confidence given by the network for each of them.

The previous definitions are illustrated on a sample example image corresponding to a street scene of Figure 7. For the sake of clarification of possible detection cases, the panels present in this scene are of a more general type (e.g., outdoor panel with the menu of a restaurant) than those in the test images.

For the previous image, one can observe that there are three panels in the scene (restaurant menu, drink and ice cream cookie, respectively). From Figure 7, one can observe that the numbers of each type of detections are TP = 1; FP = 2; TN = 1; and FN = 2.

To measure object localization accuracy, different metrics have been proposed [31,40]. The Intersection over Union (IoU) metric (also called Jaccard Index) is commonly used to evaluate the accuracy of detections and it is computed as the area of overlap between a predicted detection and its corresponding ground truth divided by the area of the union between the predicted detection and the ground truth. For binary or multi-class detection problems, the mean IoU for an image is calculated by taking the IoU of each class and averaging them. This can be extended to all the images of the test dataset to have an average IoU value.

The F1-score (also called Dice Coefficient) is another related detection metric which is calculated as two times by the area of overlap divided by the total number of pixels contained in the detected and the ground truth regions. This measure can be expressed in terms of Precision and Recall metrics. It also can be extended to all the target objects present in an image and we can compute the average F1-score for all images of the test dataset.

The IoU and F1-score metrics are related and positively correlated for a given fixed ground truth. That is, when two models are compared using IoU if the first model is better than the second one using this metric, it will also be better using F1-score. When taking the average score over a set of detections in images, the IoU metric tends to penalize quantitatively single “bad” detections more than the F1-score even when they can both agree that a given object instance is badly detected.

In order to compare our approach with the results presented by Dev et al. [21], we have included some additional average performance semantic segmentation metrics to evaluate the accuracy of detections for SSD and YOLOv3 networks. The metrics are related with pixel classification accuracy and IoU, and they are Pixel Accuracy of Class i (PA_i_), Mean Accuracy (MA), Mean IoU (M_IoU) and Frequency Weighted IoU (FW_IoU). In our case, these measures are defined for a binary detection problem (i.e., for each test image we have only the respective classes ‘panel’ and ‘no-panel’). For any test image, we denote the pixels belonging to class *i* which are predicted as belonging to class *j* as *n_ij_*, the number of pixels of class *i* is *t_i_*, and the number of classes *n_cl_* is assumed as two. Then, the new considered metrics are computed by Equations (9)–(12):

(9) $$PA_{i}=\sum_{i}\frac{n_{ii}}{t_{i}}$$

(10) $$MA=\frac{1}{n_{cl}}\sum_{i}\frac{n_{ii}}{t_{i}}$$

(11) $$M_{IoU}=\frac{1}{n_{cl}}\frac{\sum_{i}n_{ii}}{\sum_{i}\left(t_{i}+\sum_{j}n_{ji}-n_{ii}\right)}$$

(12) $$FW_{IoU}=\frac{1}{\sum_{i}t_{i}}\frac{\sum_{i}t_{i}n_{ii}}{\sum_{i}\left(t_{i}+\sum_{j}n_{ji}-n_{ii}\right)}$$

In our context, the Precision Accuracy of class ‘panel’ represents the ratio of panel pixels classified as such by the total number of pixels belonging to this class; Mean Accuracy computes average precision accuracy for classes ‘panel’ and ‘no-panel’; and Mean IoU and Frequency Weighted IoU represent measures derived from IoU that are also computed at pixel level and averaged for the set of test images. Note that all these metrics return a value between 0 and 1, where a higher value for a detection network represents a better performance.

### 3.2. Experimental Results

This subsection summarizes the quantitative and qualitative results achieved in our dataset by the two detection deep networks which are compared: SSD and YOLOv3, respectively. First, we show some global performance results for both detectors. Then, the results produced by these networks with respect to the considered variabilities are shown. Finally, these results are compared by those presented by Dev et al. [21] with respect to the same metrics described at the end of previous subsection.

#### 3.2.1. Global Performance Results.

For all our experiments, we have used 261 test images that contain a total of 283 panels. As pointed out, the values of *Conf_th_* and *IoU_th_* parameters were set to 0.5 and 0.6, respectively. Table 3 presents the global numbers of TP, FP and FN for SSD and YOLOv3. Note that for the considered panel detection problem, the concept of TN makes no sense (and consequently it is not computed).

We can conclude that both networks are able to detect correctly most of the panels: 59.4% of TP achieved by SSD (with respect to a number of 283 test panels) and 72.1% achieved by YOLOv3, respectively. It is remarkable that SSD produces a much-reduced number of FP (only three panels) but increases much more the number of FN. For the case of YOLOv3, the number of FP increases drastically while the FN are reduced to 32% with respect to SSD. In summary, SSD drastically reduces the number of false detections with respect to YOLOv3 whereas this second model produces a more reduced number of false negatives.

Table 4 presents average IoU, Precision, Recall and F1-score values achieved for SSD and YOLOv3 using the test dataset. Note that SSD produces a very high Precision result and lower Recall values in comparison with YOLOv3. This last model produces a slightly more accurate detection result with respect to IoU metric than SSD. On the other hand, SSD has a slightly higher value for F1-score. Note that F1-score tends to measure something closer to average performance, while the IoU score measures something closer to the worst-case performance.

**Figure 8:**
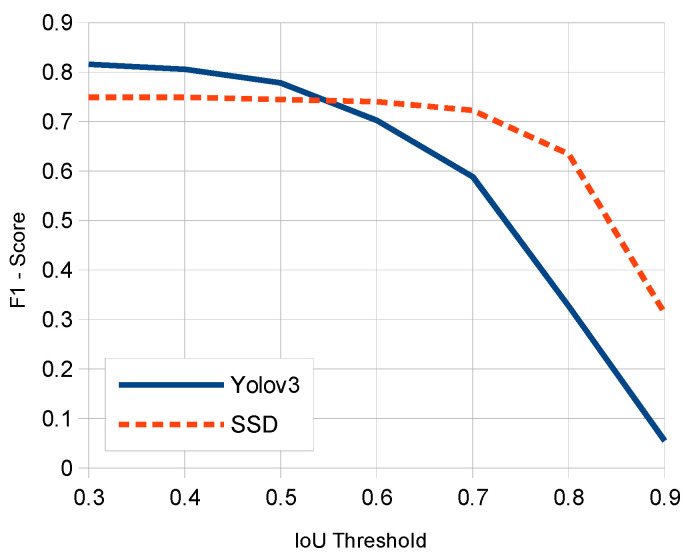
Average F1-score vs. IoU threshold comparison between SSD and YOLOv3.

Figure 8 compares respective average F1-score curves for IoU threshold values corresponding to SSD and YOLOv3 models. Note that the cutting point between the two curves (0.74 of F1-score) corresponds to an approximate IoU threshold value of 0.57. This determines the choosing of an IoU_th_ parameter value of 0.6 in our experiments. Note that for all IoU thresholds equal to or above the considered one, SSD produces a higher F1-score result.

**Figure 9:**
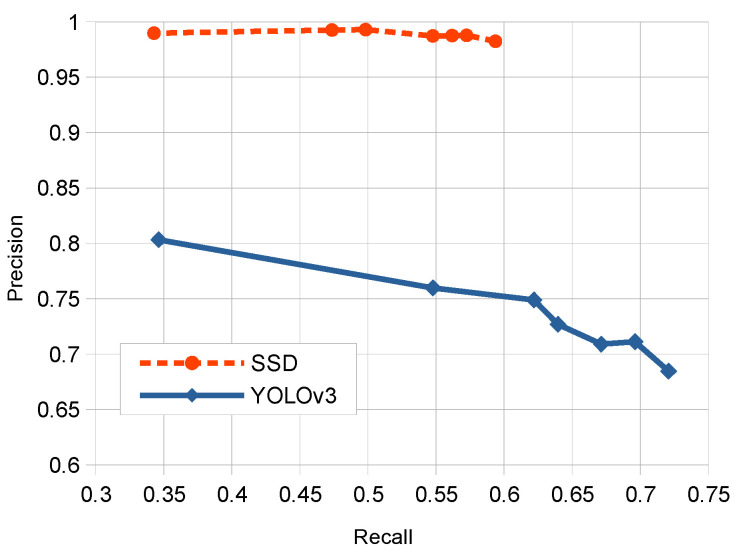
Respective precision-recall curves for SSD and YOLOv3 detectors.

Figure 9 shows the precision-recall curve that compares SSD and YOLOv3 detectors for different threshold values considered. It can be observed that due to the reduced FP value for SSD at different threshold values the precision is always very high for different recall values. On the other side, YOLOv3 presents a more reduced precision but with a higher range of corresponding recall values.

**Figure 10:**
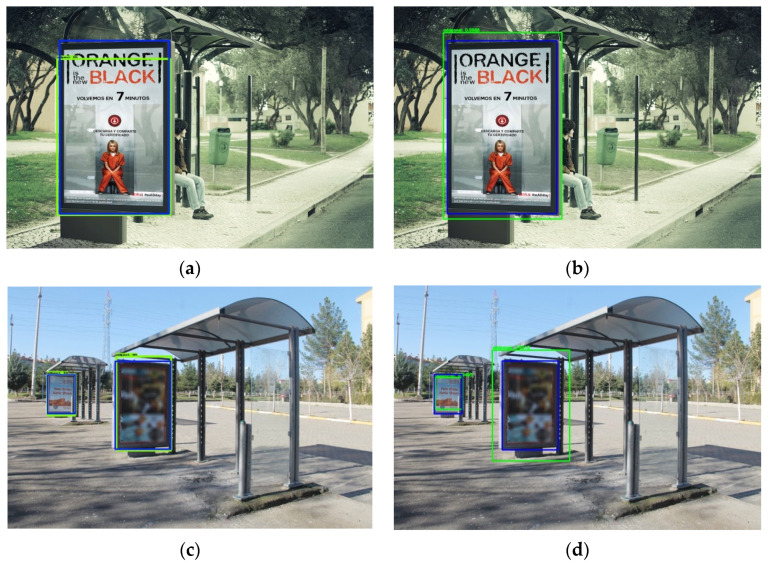
Qualitative detection results for two sample test images using SSD and YOLOv3: (**a**) SSD detection in first image; (**b**) YOLOv3 detection in first image; (**c**) SSD detection in second image; (**d**) YOLOv3 detection in second image.

Figure 10 illustrates some qualitative results corresponding to two sample test images of our dataset, where green rectangles represent detections produced by the networks and blue rectangles represent their corresponding ground truths. Each row of this Figure corresponds to the detection of a panel by SSD (left) and YOLOv3 (right). Note that the second row presents two small panels (i.e., both with a size smaller than 10% of the image) that have been correctly detected by both networks.

**Figure 11:**
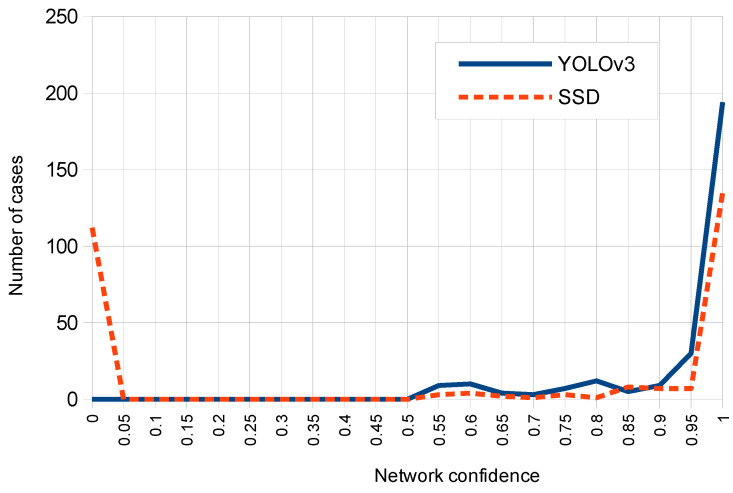
Histograms of respective network confidence distributions for SSD and YOLOv3.

Figure 11 shows the corresponding histogram for both networks which relates the confidence returned by each network with the number of test cases. Note that for YOLOv3 most cases correspond to a high confidence value between 0.95 and 1, while for SSD the most returned confidence values on detections are distributed in ranges between 0.95 and 1 (first place) and between 0 and 0.05 (second place).

**Figure 12:**
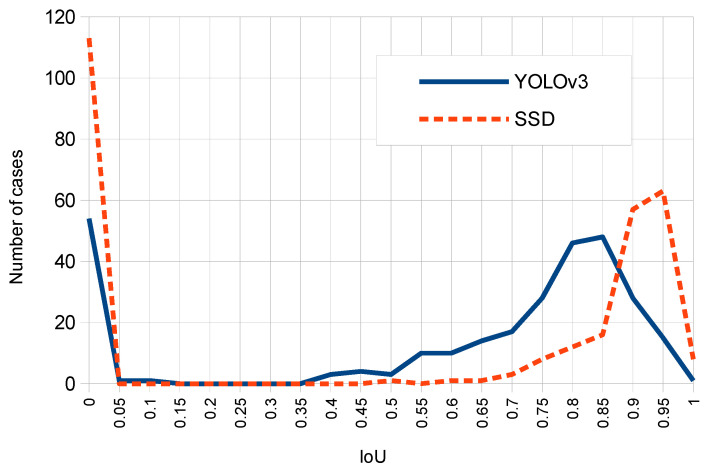
Histograms of respective IoU value distributions for SSD and YOLOv3.

Figure 12 compares SSD and YOLOv3 with respect to the number of cases for each IoU computed result. Note that although SSD produces higher IoU peaks than YOLOv3, this second network presents a higher number of TP cases (since the area of the corresponding curve above IoU threshold of 0.6 is larger for YOLOv3 compared to SSD).

#### 3.2.2. Specific Results Relative to Size of Panels in Images

This subsection analyzes the performance of each detector relative to area of panels with respect to the image size (i.e., the panel size ratio). Table 5 and Table 6, respectively, show the detection results with respect to this ratio for SSD and YOLOv3. Note that a significant number of panels (a total of 150, which corresponds to 53% of the test dataset) present a very-reduced size (i.e., the surface is smaller than 10% of the image), which makes it more difficult to detect them. Conversely, only nine panels (3.2% of them) are “big” and cover about 40% or more of the image area. The ratio TP/Panels in both tables expresses the percentage of correctly detected panels for each size, and the ratio FP/TFP is the percentage of FP with respect to total of false positives (TFP) that corresponds to each panel size.

From these two tables one can observe that YOLOv3 produces better detection results than SSD on the smallest panels (55% versus 39%), and also for the other groups of sizes (with a smaller difference). All big panels (i.e., above 40% of size ratio) are correctly detected by the two models. In general, very small panels are poorly detected by both networks. Finally, most of FP cases in YOLOv3 are produced for very small panels.

#### 3.2.3. Specific Results Relative to Panel Occlusions, Rotations and Illumination Conditions

In this subsection, we compare SSD and YOLOv3 models with respect to the other three variabilities analyzed in this study: panel occlusions and rotations (due to image formation process which maps a 3D scene into a 2D image), and scene illumination conditions, respectively. The occlusions present in the images of our dataset can reach up to 40 percent of the panel surface and rotations up to 60 degrees on the image camera plane (as illustrated by Figure 6). Table 7 and Table 8, respectively, show the distribution of images and the corresponding FN and FP detection errors for the two analyzed detectors and for each type of variability. We also present average and maximum value of IoU for each type of variability (note than minimum value of IoU is not included since it is 0 when at least one panel of the dataset is not detected).

From the two previous tables we can conclude that with respect to the panel occlusions and rotations, SSD produces very few FP (only one result in both cases). With respect to the FN under these two variabilities, the results are slightly favorable for YOLOv3. This network is much more robust than SSD under occlusions and rotations since average IoU values are, respectively, twice as good for occlusions (0.16 vs. 0.32) and around 20% better for rotations (0.46 vs. 0.55). YOLOv3 is also more robust than SSD, improving by 45% in the average IoU value when detecting panels in night images. This network present fewer FN cases, while the main advantage of SSD lies in reducing to zero the number of FP for night images.

#### 3.2.4. Comparative with Related Works

Due to the lack of datasets similar to the one used in our experiments, it is not possible to perform an exact comparison with the few related works on this topic. For such a purpose, we reproduce here the results reported by Dev et al. [21] corresponding to the outdoor advert detection problem in images using the CASE dataset (which it is not public as of yet). We have computed the same metrics in Equations (9)–(12) for SSD and YOLOv3 using our test images. Table 9 presents our results for SSD and YOLOv3 models together with those reported by Dev et al. using the FCN, PSPNet and U-Net semantic segmentation networks and these same metrics. It is remarkable that SSD and YOLOv3 produced, in general, better results with respect to pixel accuracy and IoU-derived metrics than the compared semantic segmentation networks. Moreover, YOLO3 reported the best results in two of the four metrics considered.

## 4. Discussion

**Figure 13:**
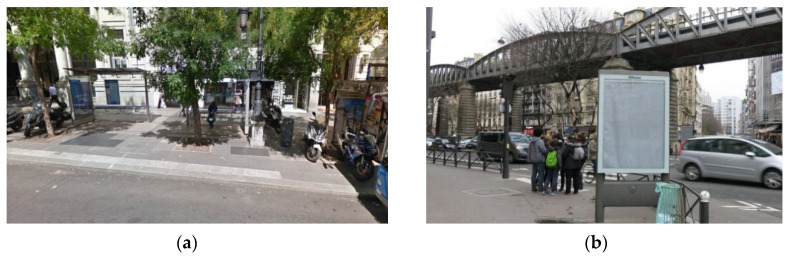
Two sample test images presenting undetected panels for SSD and YOLOv3: (**a**) complex scene; (**b**) panel without publicity.

Both detectors have been successfully able to localize most of the test panels in “difficult” conditions and combining several variabilities as is shown by the example in the second row of Figure 10. However, although these compared detectors worked well in most of test images, there exist some of them where the panels were detected neither by SSD nor by YOLOv3. Figure 13 presents two examples of undetected panels. Several combined variabilities appeared simultaneously on the left image: very small size ratio of the panel, pronounced rotation of it and the presence of shadows. On the right image, although the panel presents relatively good detection conditions (daylight, frontal and not occluded), it appears without any publicity poster. Since all the remaining panels contain a publicity advert, it seems that this “new” situation was not learnt by the two detectors and they are not able to localize the panel (i.e., these networks learned not only geometric features of panels but also the texture contained “inside the panel” to correctly detect these structures).

From the experiments and according to results produced by global metrics on test images, it can be observed that SSD is more precise than YOLOv3, since the number of FP was insignificant for the first model. On the other side, YOLOv3 was able to detect more panels than SSD (in the sense that it has produced more than 21% of TP compared to SSD) and, on average, produced slightly more accurate detections with a 15% higher IoU result. By analyzing the images, we cannot determine a specific pattern in the panels that YOLOv3 does detect but SSD does not.

Regarding specific variabilities analyzed in the images, in general, both types of networks have more difficulties when the objects being detected are very small (below 10% of the image size). YOLOv3 has a slightly better performance than SSD for all the sizes of panels (especially when they are very small). In the case of YOLOv3 most of FP errors (i.e., 88% of them) are produced by these very small panels. This network also worked better than SSD for the average IOU metric when testing partially occluded, rotated and night-illuminated panels (note that the improvement was more remarkable for the case of occlusions).

## 5. Conclusions

This paper presented a comparative study of two main one-stage object detection neural network models (SSD and YOLOv3, respectively) for the OPPI panel localization problem in outdoor images and under multiple variabilities. It should be noted that the considered problem is more challenging that classical “text in the wild” detection since there is not a predefined texture pattern (i.e., there are some panels which contain just images while others mainly contain texts). Due to the difficulty of finding annotated images for the considered problem, we created our own dataset for conducting the experiments. Both compared detectors have produced acceptable results for different panel sizes, illumination conditions, image perspective, partial occlusion of panels, complex background and multiple panels in scenes. The major strength of SSD model is the almost elimination of FP cases that is preferable in applications related to the analysis of publicity contained in the panel. On the other side, YOLOv3 produced better average detection results since it localized a higher number of TP panels and with a higher accuracy than SSD (with respect to the corresponding ground truths of test images). The study also included a comparison with semantic segmentation networks for a similar problem and under the same evaluation metrics, concluding that a similar accuracy is reached.

As future work, we aim to slightly modify the architecture of these networks to improve the detection rate accuracy for the case of very small panels. More concretely, we will investigate specifically the “difficult” images where both detection networks have failed, as is the case with the example presented in Figure 13a. We also plan to extend our work for performing the detections in indoor scene images (e.g., shopping centers or malls) where these types of panels are also available. Another interesting future work consists in recognizing the elements contained inside the panels to determine the brand names, and also to use the panel detection results to update the publicity for Augmented Reality (AR) applications. Finally, we will also study how to adapt our experiments to the new version of YOLO (i.e., YOLOv4), which has recently appeared.

## Figures and Tables

**Table 1:** Main training hyperparameters used for SSD and YOLOv3 networks.

Hyperparameter	SSD	YOLOv3
Training epochs	176,000	5000
Batch size	Between 6 and 10 images	Between 4 and 8 images
Optimizer	RMSProp	SGD Burn-In (in DarkNet [])
Learning rate	[0.001, 0.004]	[0.0001, 0.01]
Momentum	0.9	[0.8, 0.9]
Decay	0.9	[0.0003, 0.0005]

**Table 2:** Panel distribution of variabilities in the test dataset.

	Panel vs. Image Size Ratio (%)						Panel Position		Occluded		Illumination	
	≥0	≥10	≥20	≥30	≥40	≥50	Frontal	Oblique	Yes	No	Night	Day
# Panels	283	133	55	28	9	0	182	101	34	249	29	254
% Panels	100.0	47.0	19.4	9.9	3.2	0.0	64.3	35.7	12.0	88.0	10.2	89.8

**Table 3:** Respective numbers of TP, FP and FN for SSD and YOLOv3 networks.

Network	TP	FP	FN
SSD	168	3	115
YOLOv3	204	94	79

**Table 4:** Average Intersection over Union (IoU), Precision, Recall and F1-score values for SSD and YOLOv3 networks.

Network	IoU	Precision	Recall	F1-Score
SSD	0.52	0.98	0.59	0.74
YOLOv3	0.60	0.68	0.72	0.70

**Table 5:** Detection results for SSD network in relation with panel size ratios.

Size Ratio s (%)	Panels	TP	TP/Panels	FP	FP/TFP	Average IoU
0 < s < 10	150	59	0.39	1	0.33	0.52
10 ≤ s < 20	78	63	0.81	1	0.33	0.73
20 ≤ s < 30	27	22	0.81	0	0.00	0.76
30 ≤ s < 40	19	15	0.79	1	0.33	0.77
s ≥ 40	9	9	1.00	0	0.00	0.91

**Table 6:** Detection results for YOLOv3 network in relation with panel size ratios.

Size Ratio s (%)	Panels	TP	TP/Panels	FP	FP/TFP	Average IoU
0 < s < 10	150	82	0.55	83	0.88	0.60
10 ≤ s < 20	78	72	0.92	8	0.09	0.74
20 ≤ s < 30	27	24	0.88	1	0.01	0.73
30 ≤ s < 40	19	17	0.89	2	0.02	0.70
s ≥ 40	9	9	1.00	0	0.00	0.74

**Table 7:** Specific results for SSD with respect to occlusions, rotations and illumination.

Variability		# Images	FN	FP	Avg IoU	Max IoU
Panel occlusions	Yes	34	27	1	0.16	0.91
	No	249	88	2	0.57	0.97
Panel rotations	Frontal	182	67	2	0.55	0.90
	Oblique	101	48	1	0.46	0.95
Illumination	Day	254	100	97	0.53	0.97
	Night	29	15	0	0.40	0.95

**Table 8:** Specific results for YOLOv3 with respect to occlusions, rotations and illumination.

Variability		# Images	FN	FP	Avg IoU	Max IoU
Panel occlusions	Yes	34	21	22	0.32	0.87
	No	249	58	191	0.64	0.96
Panel rotations	Frontal	182	43	54	0.63	0.95
	Oblique	101	36	40	0.55	0.96
Illumination	Day	254	70	84	0.60	0.95
	Night	29	9	10	0.58	0.96

**Table 9:** Comparative with results published by Dev et al. [21], using the same metrics (best result produced for each metric appears in bold).

Network	PA (Panels)	MA	M_IoU	FW_ IoU
FCN	**0.978**	0.509	0.498	**0.959**
PSPNet	0.545	0.625	0.284	0.529
U-Net	0.619	0.727	0.327	0.601
SSD	0.956	0.835	0.749	0.931
YOLOv3	0.934	**0.872**	**0.783**	0.881
